# Supplementary material for: Activation of Pvt1b isoform contributes to local Pvt1 abundance to repress Myc during stress
Source: PLoS Genet. 2025 Jul 31;21(7):e1011790. doi: 10.1371/journal.pgen.1011790 (PMC12312954; doi:10.1371/journal.pgen.1011790)
Supplement: S1 Fig — (PDF) [file pgen.1011790.s003.pdf]

Figure S1

A

Pvt1b exon 1b sequence (g1-11 PAMs):

CTTCTTAAAGCTCTAGCCAGTGGGAAAGCGTT
TGTGTTTCTATCCTTGGAGCTCCAAGTGGGAC
TTGTTAAAAGATTTAGAAGGAGCAAAGCTGTC
AGGAAATCAGAAACGTCACATGGACTCCATGA
CTGGGAAAACCTCGTGGTGGCCTGCTCTCA
GTGCTTGGATGTGTGCTAGTTACATCTCGGAG

B

gRNA	% Mutagenesis
g1	45.6
g2	61.5
g3	N/A
g4	66.6
g5	37.2
g6	75.0
g7	90.5
g8	94.4
g9	46.5
g10	90.5
g11	79.6

C

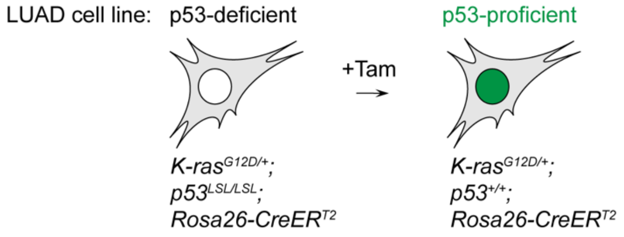

D

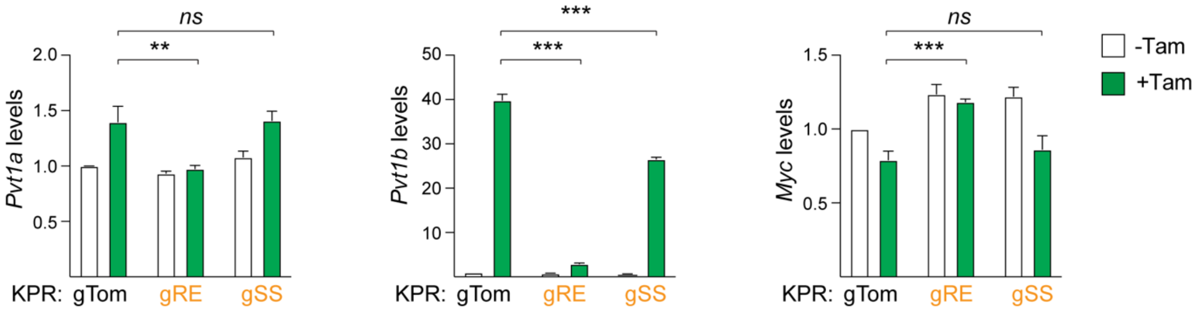

E

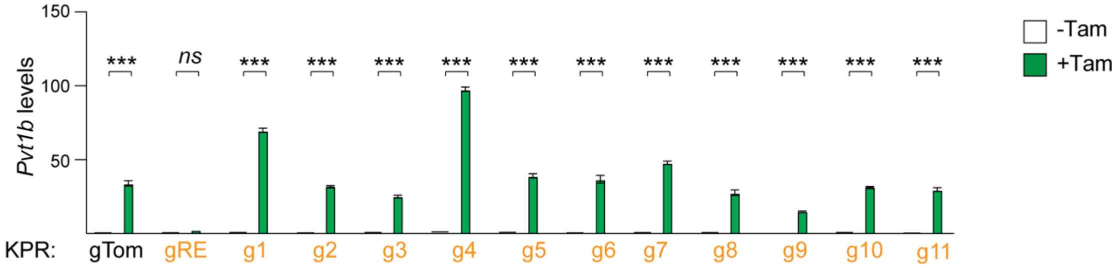

F

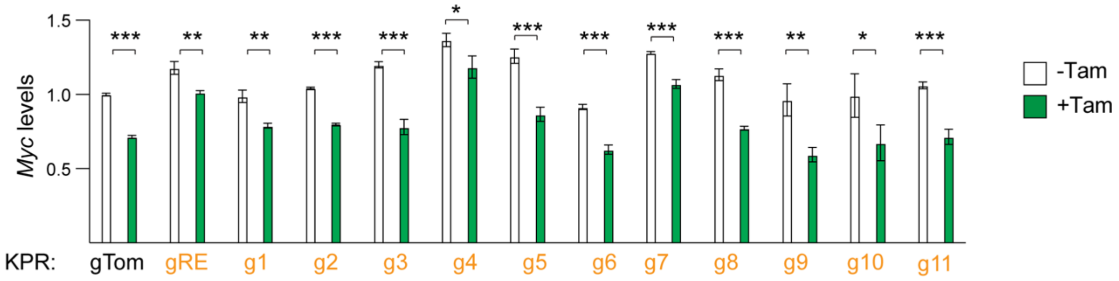

<u>Figure S1.</u> CRISPR/Cas9 screen to identify *Pvt1b*-specific functional elements in KPR cells.

A. Schematic of 11 gRNA PAM sites spanning *Pvt1* exon 1b.

B. Mutagenesis efficiency in g1-g11 KPR cells, determined as in (Tesfaye et al., 2021).

C. Schematic of $Kras^{G12D/+}$; $p53^{LSL/LSL}$; $R26\text{-}CreER^{T2}$ (KPR) lung adenocarcinoma (LUAD) cell line, showing Tamoxifen (Tam)-mediated p53 restoration and activation in the presence of oncogenic stress.

D. RT-qPCR detection of *Pvt1a*, *Pvt1b* and *Myc* RNA levels in KPR cells expressing gTom, gRE, or gSS in the absence and presence of Tam treatment. Data show mean ± SD of normalized RNA levels in n = 3 biological replicates. Paired t-test, ** p<0.01, *** p<0.001, *ns* not significant.

E, F. RT-qPCR detection of *Pvt1b* (E) and *Myc* (F) RNA levels in KPR cells expressing gTom, gRE, or g1-11 in the absence and presence of Tam treatment. Data show mean ± SD of normalized RNA levels in n = 3 technical replicates and was confirmed in an independent biological replicate.
