# Supplementary material for: Activation of Pvt1b isoform contributes to local Pvt1 abundance to repress Myc during stress
Source: PLoS Genet. 2025 Jul 31;21(7):e1011790. doi: 10.1371/journal.pgen.1011790 (PMC12312954; doi:10.1371/journal.pgen.1011790)
Supplement: Attachment — Submitted filename: Response to Reviewers.pdf [file pgen.1011790.s005.pdf]

**Response to Reviewers**

**PGENETICS-D-25-00270**

Activation of *Pvt1b* isoform contributes to local *Pvt1* abundance to repress *Myc* during stress

**Reviewer #1**

This study investigates the role of the Plasmacytoma variant translocation 1 (PVT1) lncRNA, specifically the Ptv1b isoform, in the p53-dependent repression of Myc expression following stress induction. Based upon the author's prior work demonstrating p53 targeting of Ptv1b under stress, they explore the mechanism of Myc transcriptional repression by Ptv1b, focusing on whether this effect is mediated by repressive sequence elements within exon 1b or by an increase in local Ptv1 abundance.

The authors conducted a comprehensive analysis employing well-designed in vitro and in vivo experiments with robust methodology and appropriate controls, which is a strength of the study. The use of multiple genetic mouse models further supports the findings. While acknowledging potential limitations and carefully interpreting some inconsistent data, the authors provide thorough validation of their observations across numerous experiments. The manuscript is logically structured, clearly presented, and the conclusions are well supported by the evidence data.

We are grateful to the Reviewer for the positive feedback on our model systems, methodology, and conclusions. We also greatly appreciate the specific comments provided by the Reviewer. As detailed below, we have addressed each comment directly with additional text and figure panels. In particular, we have expanded the introduction to provide more context for the study. These additions have greatly improved the strength and accessibility of the manuscript to a wider audience.

Below are some minor comments:

1. PVT1b isoform expression and regulation: Literature data demonstrate significant differential expression patterns of PVT1 isoforms across various human tissues. To provide a clearer context for the study's focus on Ptv1b, the authors could clarify the general expression differences between Ptv1a and Ptv1b. It would be valuable for the authors to discuss existing studies that indicate tissue-specific expression patterns for the Ptv1b isoform specifically.

The expression pattern of *Pvt1* isoforms is of great interest to us because of its potential relevance to Myc regulation. Existing databases, such as GTEx, indicate that *Pvt1a* expression varies in different contexts, such as during development and between tissues (see Figure below). In contrast, *Pvt1b* is not included in these datasets because of its strict dependence on p53 activity. Unfortunately, we are not familiar with reliable citation sources that discuss *Pvt1a/b* expression patterns outside of cancer. We apologize if we are omitting an important study.

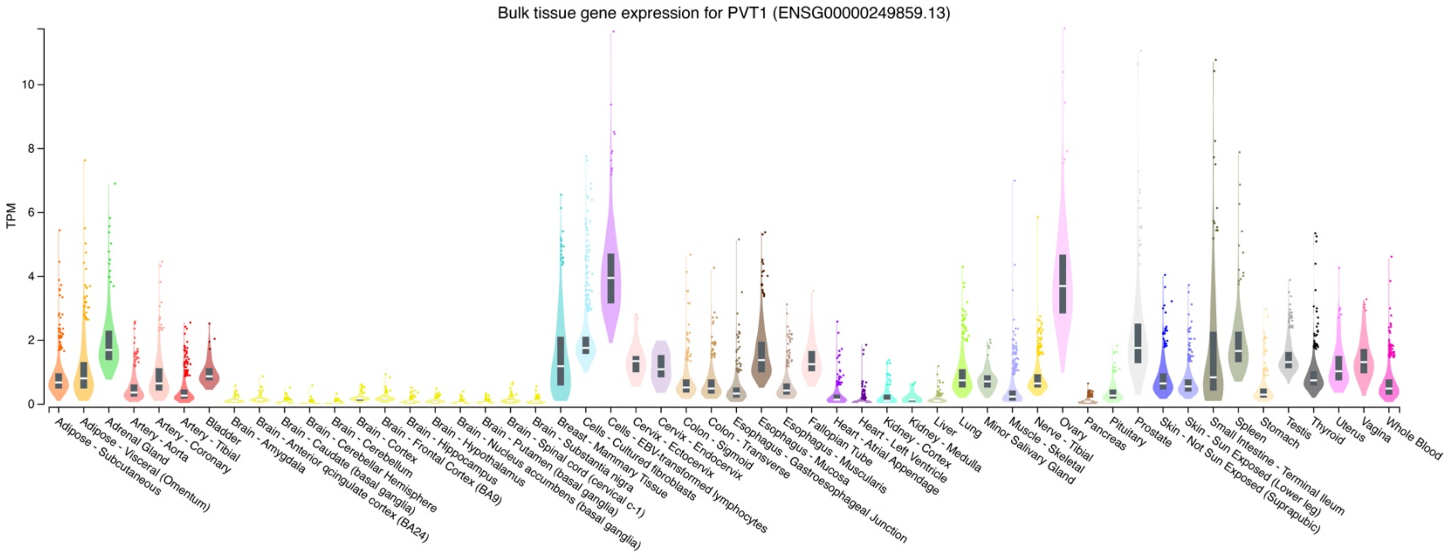


What differences were noted in the expression and abundance of Ptv1a and Ptv1b in this study? Is Ptv1a generally expressed at higher levels than Ptv1b, even following stress induction? Or were the read counts for Ptv1b greater than those for Ptv1a when stress was induced?

*Pvt1a* is the only isoform expressed in the absence of stress and remains more abundant than *Pvt1b* following stress induction. These data are presented in Figure 4B, which report on total *Pvt1a*, *Pvt1b,* and combined *Pvt1a/b* in untreated and Doxorubicin-treated MEFs. These data indicate that *Pvt1b* accounts for approximately 15% of total *Pvt1* in the presence of stress. We have specified this observation in the text.

Figure 6C, D and Supplementary Table 1 present long read counts of *Pvt1a* and Pvt1b, when expressed from *Myc/Pvt1* loci amplified in extrachromosomal DNA (ecDNA). In these data, *Pvt1b* accounts for a comparable fraction, 10-15%, of total *Pvt1*.

Furthermore, does Pvt1b have any known role in other regulatory mechanisms beyond controlling Myc expression at the transcriptional level? This information might interest the reader and provide a broader context for Ptv1b function and its regulation.

As we reported in Olivero et al., 2020, single molecular RNA FISH showed that *Pvt1b* is retained in the chromatin near its site of transcription, indicative of local activity, and RNAseq analysis revealed that loss of *Pvt1b* exclusively affects the expression of *Myc* and Myc targets. We are not familiar with studies from other groups that have shown other *Pvt1b* activities. Thus, to our knowledge, *Pvt1b* does not have any additional functions. We have expanded the introduction to emphasize these observations. We agree with the reviewer that the original introduction did not provide enough relevant background.

2. p53 binding to Ptv1b promoter: The authors' findings that p53 controls the expression of Ptv1b isoform under stress could be strengthened by citing any publicly accessible ChIP-seq data for p53 that corroborates these findings at the genomic level. This would provide an additional independent layer of evidence for their research conclusions.

Multiple p53 ChIP-seq studies in different cell lines and contexts support p53 binding in the *Pvt1* locus, suggesting that *Pvt1b* is a canonical p53 target. We have cited a select subset of these studies. Additionally, our previous published work (Olivero et al., 2020) provided comprehensive analysis of the p53-dependent regulation of *Pvt1b*, including expression data in p53-proficient and -deficient cells and p53 ChIP. We further showed that mutagenesis of the *Pvt1b*-associated p53RE abrogated p53 binding, decreased *Pvt1b* expression, and abolished *Pvt1b*-mediated *Myc* regulation during stress. In this study, we use p53RE mutagenesis

primarily as a positive control for CRISPR mutagenesis efficiency. This background is highlighted in the expanded introduction. We thank the reviewer for pointing out the omissions in our introduction.

3. Myc expression in Pvt1b-deficient mice: Do authors expect differences in Myc expression/nuclear localization in tumors from LA1 Pvt1b+/+ and LA1 Pvt1bP+/- mice? This could be potentially tested by IHC staining.

As recommended by Reviewers 1 and 2, we have performed Myc IHC in LA1; Pvt1b WT and PAS tumor-bearing mice. Quantification of the optical density of Myc staining in tumors confirmed an overall increase in Myc intensity in PAS compared to WT tumors. Even though the difference was not statistically significant ($p=0.061$), we believe that these results are consistent with our overall conclusion that *Pvt1b* loss has a moderate effect on Myc levels and tumor growth.

4. PVT1 as an oncogene and potential tumor suppressor roles: PVT1 in human is located on chromosome 8q24, a region frequently amplified in various cancers, often alongside the MYC oncogene. Therefore, PVT1 is predominantly recognized as an oncogene across a wide range of human cancers. However, authors present here evidence for tumor suppressor-like activities for Pvt1. Given the complex and context-dependent nature of lncRNA function, the authors should elaborate on the specific circumstances or cancer types where PVT1 (or potentially specific isoforms or its promoter) has been reported to exhibit tumor suppressor-like activities. This nuanced discussion would provide a more complete picture of PVT1's multifaceted role in cancer.

We thank the Reviewer for highlighting this critical question in the field and for pointing out that our introduction lacked an appropriate discussion of this topic. Recent work has led to the conclusion that 8q24 aberrations primarily serve to amplify *Myc* and its associated enhancers, several of which are located within the gene body of *Pvt1*. We do not believe that there is rigorous evidence that *Pvt1* transcripts harbor oncogenic activities. In contrast, work from us and others has emphasized the presence of tumor suppressive elements, associated with *Pvt1* transcription and transcripts. We have cited all the relevant studies that emphasize the model that while the *Pvt1* locus harbors oncogenic DNA elements (enhancers), the *Pvt1* transcripts themselves serve tumor suppressive roles by curbing *Myc* transcription.

In conclusion, the comments mentioned above are minor and do not significantly detract from the overall quality and impact of this study. I recommend this manuscript for publication.

We thank the Reviewer for their time and helpful comments.

**Reviewer #2**

We thank the review for the concise and thoughtful comments. We have addressed all the points through additional experiments, which has resulted in a stronger manuscript.

Figure 1: Add quantitative data showing the relative expression levels of Myc across all mutant lines in one comparative graph.

We have updated the graphs in Fig. 1F, G and Fig. S1 to reflect *Pvt1b* and *Myc* relative levels across all exon 1 mutants. We agree with the reviewer that this is an improved way to present the data.

Figure 2: Add a direct side-by-side quantitative comparison between Pvt1a and Pvt1b repressive activity; Include representative images of the smRNA-FISH alongside the quantification.

The repressive activity of *Pvt1a* and *Pvt1b* is measured using luciferase assay and presented on the same graph in Fig. 2E. These data reveal that *Pvt1a* and P*vt1b* have comparable repressive activity. Fig. 2C makes the point that exogenous *Pvt1a* and *Pvt1b* are expressed from sites of pTetris insertion and accumulate locally, potentially enabling local regulation of luciferase transcription. Fig. 2C is not meant to indicate regulatory activity by *Pvt1a/b*. We apologize for the confusion and have tried to improve and clarify the narrative.

Figure 4: Create a panel showing protein levels of Myc alongside RNA measurements; Enhance the senescence data with additional markers beyond β-galactosidase.

Per Reviewer's request, we have analyzed Myc protein levels in whole cell extracts from *Pvt1b* WT and PAS cells in the absence and presence of genotoxic stress. The new data, presented in Fig. 4E, F, are consistent with the RNA analysis.

We have also analyzed additional markers of senescence in *Pvt1b* WT and PAS cells treated with 0.1 μM Doxorubicin for 3 and 6 days. Consistent with the β-galactosidase data, we observe increased levels of the mitotic marker pHH3 and decreased levels of the senescence marker p16 in *Pvt1b* PAS compared to WT cells. These new analyses are presented in Fig. 4I, J.

Figure 5: Add immunohistochemistry for Myc in tumor sections to directly link the phenotype to Myc regulation; Include survival data for both genotypes to assess functional significance.

As recommended by Reviewers 1 and 2, we have performed Myc IHC in LA1; Pvt1b WT and PAS tumor-bearing mice. Quantification of the optical density of Myc staining in tumors confirmed an overall increase in Myc intensity in PAS compared to WT tumors. Even though the difference was not statistically significant ($p=0.061$), we believe that these results are consistent with our overall conclusion that *Pvt1b* loss has a moderate effect on Myc levels and tumor growth.

Consider adding a graphical abstract summarizing the overall findings.

We appreciate the suggestion. To facilitate the reader, Fig. 1A presents a schematic of the locus, highlighting *Pvt1a/b* isoforms and p53 regulatory elements. Currently, Fig. 6E presents a graphic summary of our central conclusion. We will discuss with the publication editor whether Fig. 6E might serve a better purpose as a graphical abstract.

**Reviewer #3**

Li et al. present a genetic analysis of the Pvt1 locus and its relation to Myc repression upon genotoxic stress. The work builds upon a body of prior data from the Dimitrova lab which previously showed that a spliced isoform from the Pvt1 lncRNA gene, Pvt1b, repressed Myc in a p53-dependent manner after stress. Here, Li and colleagues confirm results from those prior studies, and go on to demonstrate in a mouse model that the Pvt1b isoform plays a tumor-suppressive role in lung adenocarcinoma in vivo. At the same time, exhaustive mutational analyses demonstrate that the Pvt1b isoform per se is not required for repression of Myc, and suggest that alternate Pvt1 isoforms or overall transcription from the Pvt1 locus mediate Myc repression in a sequence-nonspecific manner. The authors interpret these data to support a model first put forth by Rick Young and colleagues, in which high local RNA levels can disrupt transcriptional condensates of a nearby promoter, in this case, Myc. The study was clearly written, elegant, and rigorously controlled. The conclusions were well supported by the data and the findings are of high significance to the field.

We thank the reviewer for the positive assessment of our work.
